# Associations Between Acute COVID-19 Symptom Profiles and Long COVID Prevalence: Population-Based Cross-Sectional Study

**DOI:** 10.2196/55697

**Published:** 2024-10-01

**Authors:** Jana L Hirschtick, Elizabeth Slocum, Yanmei Xie, Laura E Power, Michael R Elliott, Robert C Orellana, Nancy L Fleischer

**Affiliations:** 1 Department of Epidemiology, University of Michigan School of Public Health, Ann Arbor, MI, United States; 2 Advocate Aurora Research Institute, Advocate Health, 3075 Highland Parkway, Downers Grove, IL, 60515, United States, 414-219-4763; 3 Department of Biostatistics, University of Michigan School of Public Health, Ann Arbor, MI, United States; 4 Survey Research Center, Institute for Social Research, Ann Arbor, MI, United States; 5 Centers for Disease Control and Prevention Foundation, Atlanta, GA, United States; 6 Michigan Department of Health and Human Services, Lansing, MI, United States

**Keywords:** SARS-CoV-2, COVID-19, post-acute COVID-19 syndrome, epidemiology, surveillance

## Abstract

**Background:**

Growing evidence suggests that severe acute COVID-19 illness increases the risk of long COVID (also known as *post–COVID-19 condition*). However, few studies have examined associations between acute symptoms and long COVID onset.

**Objective:**

This study aimed to examine associations between acute COVID-19 symptom profiles and long COVID prevalence using a population-based sample.

**Methods:**

We used a dual mode (phone and web-based) population-based probability survey of adults with polymerase chain reaction–confirmed SARS-CoV-2 between June 2020 and May 2022 in the Michigan Disease Surveillance System to examine (1) how acute COVID-19 symptoms cluster together using latent class analysis, (2) sociodemographic and clinical predictors of symptom clusters using multinomial logistic regression accounting for classification uncertainties, and (3) associations between symptom clusters and long COVID prevalence using modified Poisson regression.

**Results:**

In our sample (n=4169), 15.9% (n=693) had long COVID, defined as new or worsening symptoms at least 90 days post SARS-CoV-2 infection. We identified 6 acute COVID-19 symptom clusters resulting from the latent class analysis, with flu-like symptoms (24.7%) and fever (23.6%) being the most prevalent in our sample, followed by nasal congestion (16.4%), multi-symptomatic (14.5%), predominance of fatigue (10.8%), and predominance of shortness of breath (10%) clusters. Long COVID prevalence was highest in the multi-symptomatic (39.7%) and predominance of shortness of breath (22.4%) clusters, followed by the flu-like symptom (15.8%), predominance of fatigue (14.5%), fever (6.4%), and nasal congestion (5.6%) clusters. After adjustment, females (vs males) had greater odds of membership in the multi-symptomatic, flu-like symptom, and predominance of fatigue clusters, while adults who were Hispanic or another race or ethnicity (vs non-Hispanic White) had greater odds of membership in the multi-symptomatic cluster. Compared with the nasal congestion cluster, the multi-symptomatic cluster had the highest prevalence of long COVID (adjusted prevalence ratio [aPR] 6.1, 95% CI 4.3‐8.7), followed by the predominance of shortness of breath (aPR 3.7, 95% CI 2.5‐5.5), flu-like symptom (aPR 2.8, 95% CI 1.9‐4.0), and predominance of fatigue (aPR 2.2, 95% CI 1.5‐3.3) clusters.

**Conclusions:**

Researchers and clinicians should consider acute COVID-19 symptom profiles when evaluating subsequent risk of long COVID, including potential mechanistic pathways in a research context, and proactively screen high-risk patients during the provision of clinical care.

## Introduction

Although SARS-CoV-2 will continue to circulate for the foreseeable future, we are now entering a new phase focused on the resulting health and social impacts of the COVID-19 pandemic. COVID-19 illness was devasting for many, resulting in over 1.1 million deaths in the United States alone [1]. Additionally, many COVID-19 survivors have not returned to their usual state of health for months or years after their COVID-19 illness. This prolonged period of recovery, commonly known as *long COVID* (also as *post–COVID-19 condition*), may affect 5% of the US adult population [2] with important implications for the health care system, workforce, and society more broadly.

There continues to be considerable uncertainty regarding long COVID, including its definition, risk factors, and mechanistic pathways. Long COVID is broadly defined as persistent new or worsening symptoms lasting for a minimum of 4 weeks (per the Centers for Disease Control and Prevention [3]) or 3 months (per the World Health Organization [4]) after a SARS-CoV-2 infection. Although there is not yet a comprehensive understanding of risk factors, a growing body of evidence reports higher long COVID prevalence among females [5-10] and individuals who had severe acute COVID-19 illness [5,6,11,12].

Acute illness course may be indicative of mechanistic pathways that operate in the development of long COVID. Relatedly, specific acute symptoms may be an important and understudied predictor of long COVID. While several studies have explored associations between acute symptoms [13-15] and long COVID prevalence, there are over 200 documented symptoms of long COVID [16] across multiple organ systems [17,18] complicating the investigatory process. Examining the clustering of acute symptoms, however, may provide more tangible information on the profiles of acute illness associated with long COVID.

We are not aware of any studies examining associations between acute symptoms and long COVID using a population-based sample. Therefore, our objectives are to examine (1) how acute COVID-19 symptoms cluster together; (2) sociodemographic and clinical predictors of symptom clusters; and (3) associations between symptom clusters and long COVID prevalence using a population-based probability survey of adults with polymerase chain reaction (PCR)–confirmed SARS-CoV-2. Findings can inform our understanding of how acute symptom profiles may predispose individuals to long COVID.

## Methods

### Study Population

Our data came from the Michigan COVID-19 Recovery Surveillance Study (MI CReSS), a population-based study of adults in Michigan with a PCR-confirmed SARS-CoV-2 infection in the Michigan Disease Surveillance System. We drew 14 sequential stratified samples of 1000 adults with COVID-19 onset between June 1, 2020, and May 31, 2022, from 13 geographic regions (6 public health preparedness regions [19], 6 counties in southeast Michigan, and Detroit). A base sample of 50 was drawn from each region, with the rest of the sample drawn proportionally to case numbers in each region during each time frame.

Eligible adults were alive at the time of sampling, not institutionalized, and had a valid phone number and zip code or county information (n=1,668,938). Of the 14,584 adults selected for the sample, 4628 provided consent and completed our survey over the phone with an interviewer in English, Spanish, or Arabic or online in English. All interviewers completed training on interviewing, the responsible conduct of research, and human subject protections prior to conducting interviews.

Of all eligible individuals in our sample, 32.2% completed the survey (American Association for Public Opinion Research response rate #6) [20]. Respondents completed the survey a median of 137 days (IQR 105‐176 days) after their COVID-19 onset date. The final sample of respondents was weighted to match the age and sex distribution within each region and the total sampling frame, simultaneously accounting for unequal probabilities of selection and nonresponse.

We excluded survey responses collected by proxy for reasons other than language translation or hearing assistance (n=19) as well as 21 phone interviews for which interviewers lacked confidence about data validity. Additionally, although there are some reports of long COVID among individuals with asymptomatic acute infection [13], we restricted our sample to individuals with acute symptoms since only 2 asymptomatic individuals out of 189 met our criteria for long COVID.

### Acute Symptom Clusters

The MI CReSS survey collected information on 27 COVID-19 symptoms. For each symptom, respondents were asked “During your illness, did you ever experience [symptom]?” If yes, respondents were asked “Did you experience this symptom during the first two weeks of your illness?” We considered an acute symptom present if the respondent experienced it in the first 2 weeks of their illness [21].

Several symptoms were collapsed into single indicators, including fever (feeling feverish or having a fever of 100.4 °F [38 °C] or above); chills (chills or repeated shaking with chills); nasal congestion (nasal congestion or runny nose); gastrointestinal symptoms (nausea or vomiting, abdominal pain, or diarrhea); and weakness (muscle weakness or general weakness). We excluded a single symptom, trouble sleeping, due to its lack of specificity. The resulting 20 symptoms were as follows: fatigue; fever; chills; muscle aches; weakness; joint pain; shortness of breath; cough; nasal congestion; sore throat; gastrointestinal; loss of appetite; headache; lightheaded or dizzy; brain fog, memory loss, or disorientation; loss of sense of smell or taste; chest pain or tightness; heart rate or heart rhythm issues; hair loss; and rash or skin discoloration. For collapsed symptoms, we coded an acute symptom present if a respondent reported at least 1 contributing symptom in the first 2 weeks of their illness. We coded an acute symptom as absent if a respondent never experienced it, did not experience it in the first 2 weeks, or did not know whether they experienced it during their illness.

### Long COVID Outcome

We defined long COVID as new or worsening symptoms lasting at least 90 days post COVID-19 onset to align with the 3-month threshold recommended by the World Health Organization [4]. We factored the time between COVID onset and the survey date into our definition since it varied across participants. Some of our respondents had less than 90 days between their COVID-19 onset and survey date. We used 2 methods (described below) to address this matter. All respondents were asked if they had recovered to their usual state of health at the time of the survey. We considered long COVID present if respondents had not recovered at least 90 days after COVID-19 onset. For respondents who had not recovered and were surveyed less than 90 days after their COVID onset, we set the long COVID variable to missing (n=56), since it is unclear what proportion of these individuals with persistent symptoms would recover by 90 days. The group identified as having long COVID remained constant in our analyses, as all of them, by definition, had 90+ days elapse between COVID-19 onset and the survey date. Alternatively, we used 2 approaches to define the group without long COVID. For the main analysis, we considered long COVID absent if respondents reported a recovery period of 90 days or less, including respondents who reported recovery who were surveyed less than 90 days after their COVID onset. We ran a sensitivity analysis excluding all individuals with less than 90 days between their COVID onset and survey date, regardless of their self-reported recovery status (n=610), to test the impact of these coding decisions.

### Covariates

We included sociodemographic and clinical factors as potential confounders, including sex at birth (male and female); age group (18‐34, 35‐44, 45‐54, 55‐64, and 65+ years); race and ethnicity (Hispanic, Non-Hispanic White, Non-Hispanic Black, another race or ethnicity); annual household income (<US $35,000, US $35,000‐74,999, and US $75,000+); and BMI (underweight/healthy weight [BMI<25], overweight [BMI 25 to <30], moderately obese [BMI 30 to <35], and severely obese [BMI 35+]). We also included a binary indicator for the presence of any of the following pre-existing diagnosed conditions: chronic obstructive pulmonary disease, asthma, diabetes, heart disease, hypertension, liver disease, kidney disease, cerebrovascular disease, cancer, immunosuppressive conditions, autoimmune conditions, or a physical disability. We coded each pre-existing condition as absent if the respondent did not know if they had ever been diagnosed with the condition. We also coded the combined pre-existing condition indicator as “none” if a respondent had missing data for 1 condition but reported no previous diagnoses for the remaining 11 conditions. We included an additional indicator for a pre-existing diagnosed psychological condition separately because prior research has found an association between a pre-existing psychological condition and long COVID [7,12,22].

### Missing Data

Data missingness was generally minimal. However, income was missing for 10.9% of respondents. To account for this, we imputed income using the hot deck imputation method under the missing at random assumption [23]. Additionally, to account for missingness in race and ethnicity (4.3%) and BMI (2%), we included an “unknown” category for each variable. The remaining missingness (4% for long COVID outcome; ≤1% for additional covariates) was handled using listwise deletion.

### Statistical Analysis

We used latent class analysis (LCA) to identify acute symptom clusters among symptomatic participants, accounting for the complex sampling design. Using the 3-step method [24,25], we (1) estimated an unconditional LCA model for acute symptom clusters, (2) assigned respondents to classes using modal posterior probabilities and calculated classification uncertainties (ie, measurement errors), and (3) explored predictors of latent class membership while accounting for these measurement errors using multinomial logistic regression. To determine the adequate number of classes, we applied unconditional LCA iteratively, beginning with a 2-class model, and sequentially increased the number of latent classes while examining the fit statistics. We evaluated goodness of fit indices by considering the Bayesian information criterion, Vuong-Lo-Mendell-Rubin likelihood ratio test, and adjusted Vuong-Lo-Mendell-Rubin likelihood ratio test. All LCA analyses were conducted using MPLUS (version 8.6) [26] and used the full information maximum likelihood method to account for missing data in latent class indicator variables.

Finally, we examined associations between the acute symptom clusters and long COVID prevalence using modified Poisson regression, adjusting for the sociodemographic and clinical factors enumerated under covariates. Poisson regression analyses accounted for the complex survey design and were performed using Stata SE (version 17.0; StataCorp) [27].

### Ethical Considerations

The MI CReSS study was deemed public health surveillance by the University of Michigan institutional review board. Respondents were mailed informed consent documents and subsequently provided consent either verbally over the phone or via the web-based survey tool.

This study, a secondary analysis of deidentified MI CReSS data, was deemed exempt by the University of Michigan institutional review board, which did not require additional consent from study subjects.

## Results

### Study Population

The analytic sample (n=4169) was predominantly female (n=2489, 54.6%) and non-Hispanic White (n=3059, 70.7%), with one-third (n=1384, 36.6%) between the ages of 18‐34 years (Table 1). Over 70% (n=2921) were overweight or obese and almost half (n=2091, 48.3%) had a pre-existing physical condition. Nearly 1 in 6 (n=693, 15.9%) had long COVID, defined as new or worsening symptoms lasting at least 90 days post SARS-CoV-2 infection.

**Table 1.** Description of the analytic sample (n=4169) from the Michigan COVID-19 Recovery Surveillance Study (June 1, 2020, to May 31, 2022).

Characteristics		Weighted percentage (%)	N
**Sex**			
	Male	45.4	1680
	Female	54.6	2489
**Age group (years)**			
	18‐34	36.6	1384
	35‐44	18.5	741
	45‐54	16.5	699
	55‐64	16.0	711
	65+	12.4	634
**Race and ethnicity**			
	Hispanic	6.6	243
	Non-Hispanic Black	7.7	292
	Another race or ethnicity	11.2	425
	Non-Hispanic White	70.7	3059
	Unknown	3.7	150
**Annual household income (US $)**			
	<35,000	28.7	1203
	35,000-74,999	30.4	1272
	75,000	40.9	1694
**Current smoking prior to illness**		9.3	402
**BMI**			
	Underweight/normal weight (BMI<25)	27.8	1194
	Overweight (BMI 25 to <30)	31.4	1293
	Moderately obese (BMI 30 to <35)	20.2	833
	Severely obese (BMI 35+)	19.2	795
	Unknown	1.4	54
**Any pre-existing physical condition (excluding psychological)**		48.3	2091
**A pre-existing psychological condition**		13.6	576
**Long COVID**		15.9	693
**Phase**			
	June 1, 2020, to September 30, 2020	13.2	535
	October 1, 2020, to February 28, 2021	22.7	924
	March 1, 2021, to May 31, 2021	13.4	534
	June 1, 2021, to September 30, 2021	14.0	561
	October 1, 2021, to February 28, 2022	19.6	795
	March 1, 2022, to May 31, 2022	17.1	820
**Survey mode**			
	Phone	32.5	1336
	Web-based	67.5	2833

### Acute Symptom Clusters

**Figure 1.**
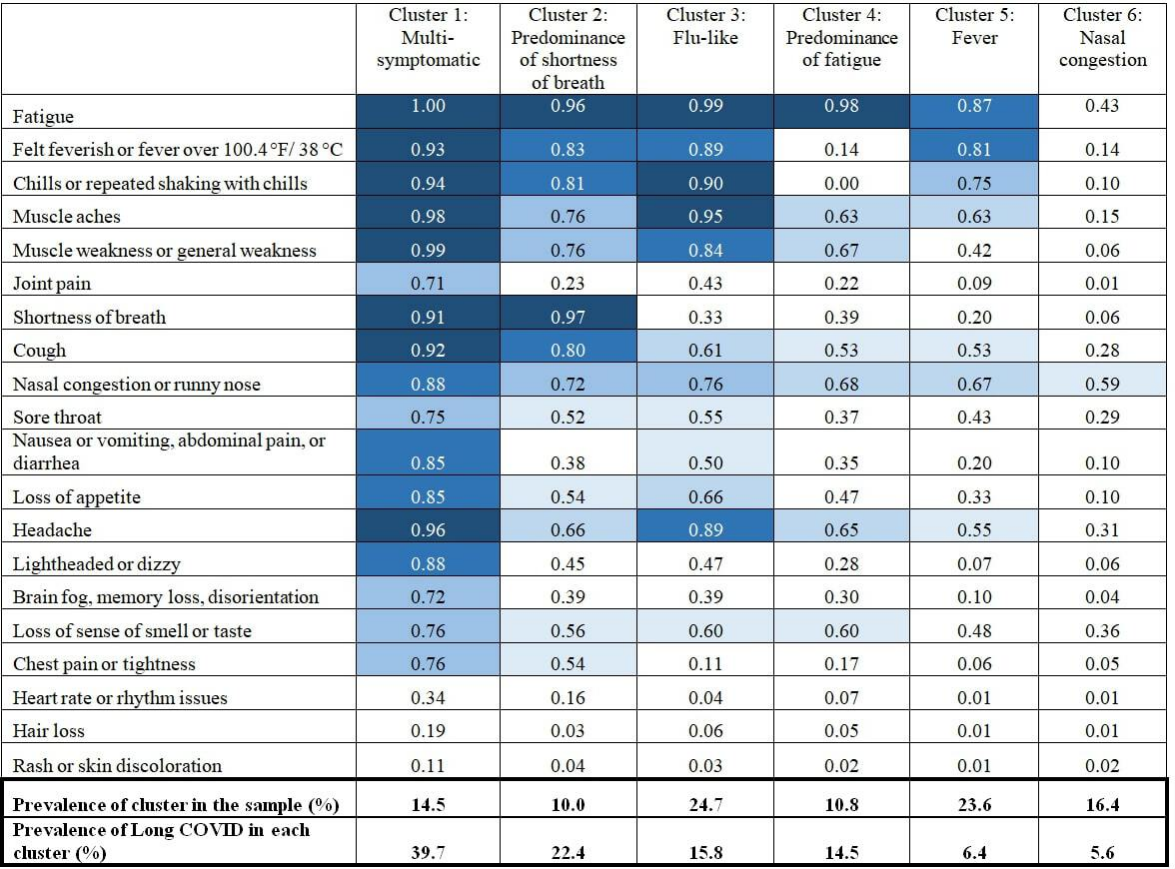
Acute COVID-19 symptom clusters in the Michigan COVID-19 Recovery Surveillance Study (June 1, 2020, to May 31, 2022). Values for symptoms represent the probability of individuals in each cluster reporting each symptom. Values with a probability of 0.5 or greater have been highlighted in blue to aid visual interpretation of findings, with darker shades of blue reflecting higher probability values.

We identified 6 mutually exclusive acute COVID-19 symptom clusters based on the Vuong-Lo-Mendell-Rubin likelihood ratio and adjusted Vuong-Lo-Mendell-Rubin likelihood ratio tests and the meaningfulness of the latent classes (Figure 1). While models with 7 classes yielded the lowest Bayesian information criterion values, the goodness-of-fit tests showed no significant difference in fit between models with 6 and 7 classes. We thus selected 6 classes based on interpretability.

Given the high probability of fatigue in 5 of the 6 classes, we did not consider it a distinguishing symptom in classes with a high probability of other symptoms. Cluster 1, representing 14.5% of the sample, was multi-symptomatic, with a high probability of flu-like (eg, fever, chills, muscle aches), pulmonary (eg, shortness of breath), gastrointestinal (eg, nausea, diarrhea), and neurological symptoms (eg, headache, dizziness). Cluster 2 (10% of the sample) had a predominance of shortness of breath. Cluster 3 (24.7% of the sample) was characterized by flu-like symptoms. Cluster 4 (10.8% of the sample) had a predominance of fatigue, without a high probability of additional symptoms. Cluster 5 (23.6% of the sample) was characterized by fever, with a lower probability of other flu-like symptoms than clusters 1 or 2. Cluster 6 (16.4% of the sample) had a low probability of all symptoms except nasal congestion.

The unadjusted (Table S1 in Multimedia Appendix 1) and adjusted results (Table 2) from the multinomial logistic regression models further characterize the acute symptom clusters. Compared with the nasal congestion cluster, females had greater odds of membership in the multi-symptomatic (adjusted odds ratio [aOR] 2.7, 95% CI 2.0‐3.8), flu-like (aOR 2.1, 95% CI 1.6‐2.8), and predominance of fatigue (aOR 1.8, 95% CI 1.2‐2.6) symptom clusters than males. Adults aged 65 years and older had lower odds of membership in the multi-symptomatic, predominance of shortness of breath, flu-like, and fever symptom clusters than adults aged 18‐34 years. However, adults aged 35‐54 years had greater odds of predominance of fatigue than adults aged 18‐34 years. Compared with Non-Hispanic White adults, adults identifying as Hispanic (aOR 2.0, 95% CI 1.1‐3.5) or another race or ethnicity (aOR 2.0, 95% CI 1.2‐3.2) had greater odds of membership in the multi-symptomatic cluster versus the nasal congestion cluster.

**Table 2.** Adjusted multinomial logistic regression examining odds of belonging to specific acute COVID-19 symptom clusters (referent category: nasal congestion; n=4169) in the Michigan COVID-19 Recovery Surveillance Study (June 1, 2020, to May 31, 2022). All models mutually adjusted for variables listed in the table. Italicized values denote statistical significance at *P*<.05.

		Multi-symptomatic				Predominance of shortness of breath				Flu-like				Predominance of fatigue				Fever			
		95% CI			*P* value	95% CI			*P* value	95% CI			*P* value	95% CI			*P* value	95% CI			*P* value
		aOR^a^	LB^b^	UB^c^		aOR	LB	UB		aOR	LB	UB		aOR	LB	UB		aOR	LB	UB	
**Sex**																					
	Male	1.0	—^d^	—	—	1.0	—	—	—	1.0	—	—	—	1.0	—	—	—	1.0	—	—	—
	Female	*2.7*	*2.0*	*3.8*	*<.001*	1.1	0.7	1.6	.64	*2.1*	*1.6*	*2.8*	*<.001*	*1.8*	*1.2*	*2.6*	*.002*	0.8	0.6	1.1	.23
**Age group (years)**																					
	18‐34	1.0	—	—	—	1.0	—	—	—	1.0	—	—	—	1.0	—	—	—	1.0	—	—	—
	35‐44	1.1	0.7	1.7	.63	0.9	0.5	1.5	.60	1.1	0.7	1.6	.69	*2.5*	*1.4*	*4.4*	*.001*	1.0	0.6	1.5	.95
	45‐54	1.2	0.8	1.9	.43	0.9	0.5	1.6	.74	1.1	0.7	1.7	.64	*1.9*	*1.0*	*3.6*	*.049*	1.4	0.9	2.2	.19
	55‐64	0.6	0.4	1.0	.07	0.7	0.4	1.3	.27	0.7	0.5	1.1	.09	1.7	1.0	3.1	.07	1.0	0.6	1.6	.99
	65+	*0.3*	*0.2*	*0.5*	*<.001*	*0.3*	*0.2*	*0.6*	*.001*	*0.3*	*0.2*	*0.5*	*<.001*	1.6	0.9	2.8	.12	*0.5*	*0.3*	*0.8*	*.003*
**Race and ethnicity**																					
	Hispanic	*2.0*	*1.1*	*3.5*	*.019*	0.7	0.3	1.6	.35	0.7	0.4	1.3	.21	1.0	0.5	2.0	.94	0.9	0.5	1.7	.68
	Non-Hispanic Black	0.9	0.5	1.6	.76	0.9	0.5	1.8	.86	0.8	0.5	1.3	.38	0.5	0.2	1.1	.07	0.6	0.3	1.2	.14
	Another race or ethnicity	*2.0*	*1.2*	*3.2*	*.007*	1.3	0.7	2.5	.35	1.1	0.7	1.8	.69	0.8	0.4	1.7	.59	1.2	0.7	2.0	.52
	Non-Hispanic White	1.0	—	—	—	1.0	—	—	—	1.0	—	—	—	1.0	—	—	—	1.0	—	—	—
	Unknown	1.7	0.7	4.0	.20	1.3	0.5	3.4	.62	1.0	0.5	2.1	.98	1.1	0.4	3.0	.84	0.9	0.4	2.1	.82
**Annual income (US $)**																					
	<35,000	*2.2*	*0.7*	*4.0*	*<.001*	*2.5*	*1.5*	*4.1*	*<.001*	1.3	0.9	1.9	.11	*1.6*	*1.0*	*2.6*	*.046*	1.3	0.9	1.9	.23
	35,000-74,999	*1.5*	*1.0*	*2.2*	*.03*	1.5	0.9	2.5	.08	1.1	0.8	1.5	.62	1.3	0.8	2.0	.25	1.0	0.7	1.4	.83
	75,000	1.0	—	—	—	1.0	—	—	—	1.0	—	—	—	1.0	—	—	—	1.0	—	—	—
**Smoking prior to illness**		*1.7*	*1.0*	*2.8*	*.04*	0.9	0.4	1.9	.82	1.4	0.8	2.2	.23	1.3	0.7	2.5	.43	1.0	0.6	1.8	.97
**BMI**																					
	Underweight/normal weight	1.0	—	—	—	1.0	—	—	—	1.0	—	—	—	1.0	—	—	—	1.0	—	—	—
	Overweight	1.2	0.8	1.9	.30	1.5	0.9	2.7	.12	*1.6*	*1.1*	*2.3*	*.009*	1.2	0.7	1.9	.46	1.2	0.8	1.8	.28
	Moderately obese	1.6	1.0	2.5	.05	*2.0*	*1.1*	*3.6*	*.02*	*1.5*	*1.0*	*2.3*	*.04*	1.4	0.8	2.3	.21	1.0	0.7	1.6	.96
	Severely obese	1.6	1.0	2.5	.05	1.6	0.9	2.9	.10	1.2	0.8	1.9	.33	1.1	0.6	1.8	.86	1.0	0.6	1.6	.93
	Unknown	1.1	0.3	4.6	.87	2.0	0.4	8.9	.38	1.2	0.3	4.8	.81	1.4	0.3	6.5	.71	1.6	0.4	5.9	.51
**Pre-existing physical condition** ^**e**^		*2.3*	*1.6*	*3.2*	*<.001*	*2.1*	*1.4*	*3.2*	*<.001*	*1.6*	*1.2*	*2.2*	*.003*	*1.6*	*1.1*	*2.4*	*.02*	1.1	0.8	1.5	.77
**Pre-existing psychological condition**		*3.3*	*2.1*	*5.2*	*<.001*	1.6	0.9	3.0	.12	*1.9*	*1.2*	*2.9*	*.007*	1.4	0.7	2.6	.31	0.9	0.5	1.6	.69
**Survey phase**																					
	June 1, 2020, to September 30, 2020	1.0	—	—	—	1.0	—	—	—	1.0	—	—	—	1.0	—	—	—	1.0	—	—	—
	October 1, 2020, to February 28, 2021	1.1	0.7	1.9	.68	*0.5*	*0.3*	*1.0*	*.04*	1.5	0.9	2.3	.12	0.9	0.5	1.6	.64	0.9	0.5	1.5	.63
	March 1, 2021, to May 31, 2021	1.7	0.9	3.0	.09	0.9	0.5	1.7	.66	1.5	0.9	2.7	.13	0.8	0.4	1.5	.44	1.0	0.6	1.9	.93
	June 1, 2021, to September 30, 2021	1.2	0.6	2.2	.61	0.7	0.4	1.4	.36	1.5	0.9	2.5	.16	1.0	0.5	2.0	.94	1.4	0.8	2.5	.21
	October 1, 2021, to February 28, 2022	1.5	0.9	2.5	.15	0.6	0.3	1.2	.14	1.4	0.9	2.3	.18	0.8	0.4	1.5	.52	1.2	0.7	2.1	.50
	March 1, 2022, to May 31, 2022	0.8	0.4	1.5	.48	0.5	0.3	1.0	.06	1.3	0.8	2.2	.27	0.8	0.4	1.4	.39	1.6	1.0	2.7	.07
**Survey mode**																					
	Phone	1.3	0.9	1.8	.14	1.0	0.7	1.6	.95	0.9	0.7	1.3	.66	1.1	0.8	1.7	.56	0.7	0.5	1.0	.07
	Web-based	1.0	—	—	—	1.0	—	—	—	1.0	—	—	—	1.0	—	—	—	1.0	—	—	—

a aOR: adjusted odds ratio.

b LB: lower bound of 95% CI.

c UB: upper bound of 95% CI.

d Not applicable (referent value).

e Excluding psychological conditions.

Annual household income and health status indicators, including BMI and pre-existing conditions, were strong predictors of symptom cluster membership. Compared with the nasal congestion cluster, adults with a household income less than US $35,000 (vs US $75,000+) had between 1.6‐2.5 times greater odds of membership in the multi-symptomatic, predominance of shortness of breath, and predominance of fatigue symptom clusters in adjusted models. In terms of health status, adults who were obese had greater odds of membership in the predominance of shortness of breath and flu-like symptom clusters. Additionally, having a pre-existing physical condition (vs none) was associated with greater odds of membership in the multi-symptomatic, predominance of shortness of breath, flu-like, and predominance of fatigue symptom clusters compared with the nasal congestion cluster. Lastly, adults with a pre-existing psychological condition (vs none) had greater odds of membership in the multi-symptomatic (aOR 3.3, 95% CI 2.1‐5.2) and flu-like symptom (aOR 1.9, 95% 1.2‐2.9) clusters.

### Long COVID Outcome

Long COVID prevalence varied across the symptom clusters, from 39.7% in the multi-symptomatic cluster to 5.6% in the nasal congestion cluster (Figure 1). In adjusted models (Table 3), the multi-symptomatic cluster had the highest prevalence of long COVID compared with the nasal congestion cluster (adjusted prevalence ratio [aPR] 6.1, 95% CI 4.3‐8.7), followed by predominance of shortness of breath (aPR 3.7, 95% CI 2.5‐5.5), flu-like symptoms (aPR 2.8, 95% CI 1.9‐4.0), and predominance of fatigue (aPR 2.2, 95% CI 1.5‐3.3). The prevalence of long COVID was not statistically different between the clusters characterized by fever and nasal congestion (aPR 1.3, 95% CI 0.8‐1.9). Sociodemographic and health-related predictors of long COVID included female sex, older age, lower income, obesity, and pre-existing conditions. Responding via telephone (vs online) was also statistically associated with higher long COVID prevalence, likely due to associations between survey mode and respondent demographics (data not shown) and potential differences in symptoms reported based on mode.

Finally, results from the sensitivity analysis excluding all individuals with less than 90 days between their COVID onset and survey date closely aligned with the primary analysis, including the 6 acute symptom clusters identified (Table S2 in Multimedia Appendix 1) and their relationship with long COVID prevalence (Table S3 in Multimedia Appendix 1).

**Table 3.** Modified Poisson regression examining prevalence of long COVID (n=4169) in the Michigan COVID-19 Recovery Surveillance Study (June 1, 2020, to May 31, 2022). Italicized values denote statistical significance at *P*<.05.

		Unadjusted				Adjusted^a^			
		95% CI			*P* value	95% CI			*P* value
		PR^b^	LB^c^	UB^d^		aPR^e^	LB	UB	
**Acute COVID-19 symptom clusters**									
	Multi-symptomatic	*7.1*	*5.0*	*10.2*	*<.001*	*6.1*	*4.3*	*8.7*	*<.001*
	Predominance of shortness of breath	*4.0*	*2.7*	*6.0*	*<.001*	*3.7*	*2.5*	*5.5*	*<.001*
	Flu-like	*2.8*	*2.0*	*4.1*	*<.001*	*2.8*	*1.9*	*4.0*	*<.001*
	Predominance of fatigue	*2.6*	*1.7*	*3.9*	*<.001*	*2.2*	*1.5*	*3.3*	*<.001*
	Fever	1.2	0.8	1.8	.51	1.3	0.8	1.9	.27
	Nasal congestion	1.0	—^f^	—	—	1.0	—	—	—
**Sex**									
	Male	1.0	—	—	—	1.0	—	—	—
	Female	*1.7*	*1.4*	*2.0*	*<.001*	*1.4*	*1.2*	*1.7*	*<.001*
**Age group (years)**									
	18‐34	1.0	—	—	—	1.0	—	—	—
	35‐44	*1.8*	*1.4*	*2.3*	*<.001*	*1.7*	*1.4*	*2.2*	*<.001*
	45‐54	*1.8*	*1.4*	*2.3*	*<.001*	*1.7*	*1.4*	*2.2*	*<.001*
	55‐64	*1.9*	*1.5*	*2.4*	*<.001*	*2.1*	*1.7*	*2.6*	*<.001*
	65+	*2.2*	*1.7*	*2.8*	*<.001*	*2.5*	*1.9*	*3.1*	*<.001*
**Race and ethnicity**									
	Hispanic	*1.5*	*1.1*	*1.9*	*.003*	1.1	0.9	1.4	.44
	Non-Hispanic Black	*1.5*	*1.2*	*1.9*	*.002*	1.1	0.9	1.5	.28
	Another race or ethnicity	0.9	0.7	1.2	.36	0.8	0.6	1.1	.15
	Non-Hispanic White	1.0	—	—	—	1.0	—	—	—
	Unknown	1.0	0.6	1.5	.85	1.0	0.6	1.5	.88
**Annual income (US $)**									
	<35,000	*1.7*	*1.4*	*2.1*	*<.001*	*1.3*	*1.0*	*1.5*	*.015*
	35,000-74,999	*1.4*	*1.2*	*1.7*	*.001*	*1.2*	*1.0*	*1.5*	*.047*
	75,000	1.0	—	—	—	1.0	—	—	—
**Smoking prior to illness**		*1.3*	*1.0*	*1.6*	*.05*	1.1	0.8	1.3	.65
**BMI**									
	Underweight/normal weight	1.0	—	—	—	1.0	—	—	—
	Overweight	*1.3*	*1.0*	*1.6*	*.03*	1.1	0.9	1.4	.33
	Moderately obese	*1.6*	*1.3*	*2.0*	*<.001*	1.2	0.9	1.5	.16
	Severely obese	*2.0*	*1.6*	*2.5*	*<.001*	*1.3*	*1.1*	*1.7*	*.01*
	Unknown	1.2	0.6	2.5	.60	1.0	0.5	2.1	.94
**Pre-existing physical condition^g^** **versus none**		*1.8*	*1.5*	*2.1*	*<.001*	*1.2*	*1.0*	*1.4*	*.05*
**Pre-existing psychological condition versus none**		*1.5*	*1.2*	*1.8*	*<.001*	1.1	0.9	1.3	.29
**Survey phase**									
	June 1, 2020, to September 30, 2020	1.0	—	—	—	1.0	—	—	—
	October 1, 2020, to February 28, 2021	1.0	0.8	1.2	.78	0.9	0.7	1.1	.37
	March 1, 2021, to May 31, 2021	0.9	0.7	1.1	.26	*0.8*	*0.6*	*1.0*	*.025*
	June 1, 2021, to September 30, 2021	*0.7*	*0.5*	*0.9*	*.005*	*0.7*	*0.5*	*0.9*	*.002*
	October 1, 2021, to February 28, 2022	*0.8*	*0.6*	*1.0*	*.04*	*0.7*	*0.5*	*0.8*	*<.001*
	March 1, 2022, to May 31, 2022	*0.3*	*0.2*	*0.4*	*<.001*	*0.4*	*0.3*	*0.5*	*<.001*
**Survey mode**									
	Phone	*1.6*	*1.4*	*1.9*	*<.001*	*1.3*	*1.1*	*1.5*	*.004*
	Web-based	1.0	—	—	—	1.0—	—	—	—

a All models mutually adjusted for variables listed in table.

b PR: prevalence ratio.

c LB: lower bound of 95% CI.

d UB: upper bound of 95% CI.

e aPR: adjusted prevalence ratio.

f Not applicable (referent value).

g Excluding psychological conditions.

## Discussion

Using a population-based sample of adults with PCR-confirmed SARS-CoV-2 in Michigan, we examined associations between acute COVID-19 symptom clusters and long COVID. Generally, we found that long COVID prevalence increased as the number of acute symptoms, or probability of severe symptoms, increased. Of the 6 acute symptom clusters we identified, long COVID prevalence was lowest in clusters with a low number of symptoms (nasal congestion, fever), and highest in clusters with more symptoms (multi-symptomatic) or a high probability of severe symptoms (shortness of breath). These findings are consistent with a growing body of research linking severe acute illness to long COVID [5,6,11,12].

Long COVID prevalence was greater among respondents with multi-symptomatic acute illness with a high probability of fever, chills, and muscle aches. This is consistent with a study of patients at a hospital in Toronto, Canada, in which patients with acute constitutional (eg, fever, chills) or rheumatologic symptoms (eg, muscle aches) had greater odds of persistent symptoms 90 days after illness onset than patients without these acute symptoms [15]. Other studies have used a different approach, examining the progression of specific symptoms during and after COVID-19 illness [16,28], rather than associations between acute symptoms and long COVID (defined as the presence of any symptom after a specified time period). For example, a study using an international convenience sample of individuals with persistent symptoms following COVID-19 illness reported 3 clusters of COVID-19 symptoms, including symptoms most prevalent during the acute phase (eg, diarrhea and fever), symptoms consistently present over time (eg, muscle aches, changes to sense of taste and smell, and fatigue), and symptoms increasingly prevalent over time (eg, brain fog and postexertional malaise) [16].

In terms of specific acute COVID-19 symptoms, our findings may provide clues to potential mechanisms leading to long COVID. The multi-symptomatic cluster had the highest probability of acute gastrointestinal symptoms. Although few studies have examined associations between acute symptoms and long COVID, one small study in India found an association between diarrhea during acute illness and long COVID [14]. There is also preliminary evidence that the SARS-CoV-2 virus persists in the gastrointestinal tract for months in a subset of infected individuals [29], potentially prolonging gastrointestinal symptoms or leading to symptoms outside the gastrointestinal tract [30].

The multi-symptomatic acute symptom cluster also had a high probability of neurological symptoms and, along with the shortness of breath cluster, respiratory symptoms. In a study of military health system beneficiaries, individuals with neurological and respiratory/systemic symptoms were more likely to report persistent symptoms 6 months following illness onset than participants with nasal symptoms [31]. Neurological and respiratory symptoms are common among individuals with long COVID [32,33], suggesting the persistence of these symptoms over time. However, neurological symptoms associated with acute COVID-19 illness and long COVID may differ. While loss of taste and smell may persist from acute to chronic illness, other neurological long COVID symptoms, such as absent-mindedness, may have a delayed onset [16,34,35], suggesting a separate mechanistic pathway.

We also identified sociodemographic disparities in acute COVID-19 symptom class membership. Since long COVID may be underdiagnosed among people who are from racial or ethnic minority groups or have low income [36], it is important to identify high-risk groups when attempting to quantify disparities in long COVID. Respondents who were female, younger, members of racial or ethnic minority groups, low income, and diagnosed with pre-existing physical or psychological conditions had greater odds of membership in the multi-symptomatic cluster, which had the highest long COVID prevalence (39.7%). Similarly, respondents who were younger, low income, and diagnosed with a pre-existing physical condition had greater odds of membership in the predominance of shortness of breath cluster, which had a long COVID prevalence of 22.4%. On the other hand, respondents who were female and middle-aged had greater odds of membership in the predominance of fatigue cluster, which had a long COVID prevalence of 14.5%.

There are also alternative explanations for these associations. First, residual confounding due to sociodemographic and health status differences may account for some of the association between the acute symptom clusters and long COVID. Second, we did not adjust for hospital admission since it likely mediates the relationship between acute symptoms and long COVID. However, post-hospital or post-ICU syndrome, whereby hospitalized individuals have prolonged recovery irrespective of admitting illness [37,38], may partially explain associations between the more severe acute symptom clusters and long COVID.

Additionally, these findings should be interpreted within the context of several limitations. First, our sample is based on adults with PCR-confirmed SARS-CoV-2 in Michigan and therefore only representative of individuals who obtained a SARS-CoV-2 PCR test in Michigan. Given the limited availability of PCR testing early on, and the wide availability of at-home testing in later stages of the pandemic, our sample is not generalizable to all individuals with SARS-CoV-2 in Michigan during our study period. Second, our response rate (32.2%), although fairly high for a population-based survey, may be subject to nonresponse bias. However, population-based samples are more generalizable than clinical or convenience samples and we have previously shown that the impact of nonresponse on our study findings is likely minimal [39]. Third, our study relies on self-reported data, which is subject to recall and reporting biases. Fourth, we did not adjust for acute illness severity, including hospital admission, as we viewed these factors as mediators between acute symptoms and long COVID. Fifth, we did not examine the persistence of specific symptoms over time, an important avenue for future research.

A growing body of research links acute COVID-19 severity to long COVID. In our population-based sample of adults with PCR-confirmed SARS-CoV-2, we found that acute COVID-19 symptoms clustered into 6 classes ranging in symptom type and severity and that long COVID prevalence increased as the number of acute symptoms, or probability of severe symptoms, increased. Researchers and clinicians should consider acute COVID-19 symptom profiles when evaluating subsequent risk of long COVID, including potential mechanistic pathways in a research context, and proactively screen high-risk patients during the provision of clinical care.

## Supplementary material

10.2196/55697 Multimedia Appendix 1 Supplementary tables containing the unadjusted results from the multinomial logistic regression models and the results from the sensitivity analysis excluding all individuals with less than 90 days between their COVID onset and survey date.

## Abbreviations

aOR: adjusted odds ratio
aPR: adjusted prevalence ratio
LCA: latent class analysis
MI CReSS: Michigan COVID-19 Recovery Surveillance Study
PCR: polymerase chain reaction
