# Supplementary material for: Associations Between Acute COVID-19 Symptom Profiles and Long COVID Prevalence: Population-Based Cross-Sectional Study
Source: JMIR Public Health Surveill. 2024 Oct 1;10:e55697. doi: 10.2196/55697 (PMC11460306; doi:10.2196/55697)
Supplement: Multimedia Appendix 1 [file publichealth-v10-e55697-s001.docx]

Appendix Table 1. Unadjusted multinomial logistic regression examining odds of belonging to specific acute COVID-19 symptom clusters (referent category: nasal congestion; n=4169), Michigan COVID-19 Recovery Surveillance Study (June 1, 2020 - May 31, 2022)

|  | Multi-symptomatic | | | | Predominance of shortness of breath | | | | Flu-like | | | | Predominance of fatigue | | | | Fever | | | |
| --- | --- | --- | --- | --- | --- | --- | --- | --- | --- | --- | --- | --- | --- | --- | --- | --- | --- | --- | --- | --- |
|  | OR | 95 % CI | | p value | OR | 95 % CI | | p value | OR | 95 % CI | | p value | OR | 95 % CI | | p value | OR | 95 % CI | | p value |
|  |  | LB | UB |  |  | LB | UB |  |  | LB | UB |  |  | LB | UB |  |  | LB | UB |  |
| Sex |  |  |  |  |  |  |  |  |  |  |  |  |  |  |  |  |  |  |  |  |
| Male | 1.0 |  |  |  | 1.0 |  |  |  | 1.0 |  |  |  | 1.0 |  |  |  | 1.0 |  |  |  |
| Female | **2.8** | **2.0** | **3.8** | **0.000** | 1.1 | 0.8 | 1.6 | 0.621 | **2.1** | **1.6** | **2.7** | **0.001** | **1.8** | **1.3** | **2.6** | **0.001** | 0.8 | 0.6 | 1.1 | 0.248 |
| Age group |  |  |  |  |  |  |  |  |  |  |  |  |  |  |  |  |  |  |  |  |
| 18-34 | 1.0 |  |  |  | 1.0 |  |  |  | 1.0 |  |  |  | 1.0 |  |  |  | 1.0 |  |  |  |
| 35-44 | 1.1 | 0.7 | 1.7 | 0.618 | 0.9 | 0.5 | 1.5 | 0.626 | 1.2 | 0.8 | 1.8 | 0.360 | **2.7** | **1.5** | **4.6** | **0.001** | 1.0 | 0.6 | 1.5 | 0.856 |
| 45-54 | 1.3 | 0.8 | 2.0 | 0.232 | 1.0 | 0.5 | 1.7 | 0.874 | 1.3 | 0.9 | 2.0 | 0.156 | **2.2** | **1.2** | **4.1** | **0.010** | 1.4 | 0.9 | 2.1 | 0.168 |
| 55-64 | 0.7 | 0.4 | 1.0 | 0.065 | 0.8 | 0.5 | 1.3 | 0.354 | 0.8 | 0.5 | 1.2 | 0.262 | **2.1** | **1.2** | **3.7** | **0.009** | 1.1 | 0.7 | 1.6 | 0.740 |
| 65+ | **0.3** | **0.2** | **0.5** | **0.000** | **0.5** | **0.3** | **0.8** | **0.009** | **0.4** | **0.3** | **0.6** | **0.000** | **2.3** | **1.3** | **3.8** | **0.002** | **0.5** | **0.3** | **0.8** | **0.003** |
| Race/ethnicity |  |  |  |  |  |  |  |  |  |  |  |  |  |  |  |  |  |  |  |  |
| Hispanic | **2.5** | **1.5** | **4.3** | **0.000** | 0.9 | 0.4 | 2.1 | 0.808 | 0.7 | 0.4 | 1.4 | 0.337 | 1.0 | 0.5 | 2.1 | 0.904 | 0.9 | 0.5 | 1.7 | 0.756 |
| Non-Hispanic Black | 1.6 | 1.0 | 2.6 | 0.066 | 1.4 | 0.8 | 2.6 | 0.235 | 1.0 | 0.6 | 1.6 | 0.952 | 0.5 | 0.2 | 1.1 | 0.103 | 0.7 | 0.4 | 1.2 | 0.193 |
| Another race/ethnicity | **2.2** | **1.4** | **3.5** | **0.001** | 1.6 | 0.9 | 2.9 | 0.124 | 1.1 | 0.7 | 1.8 | 0.606 | 0.8 | 0.4 | 1.5 | 0.452 | 1.3 | 0.8 | 2.2 | 0.247 |
| Non-Hispanic white | 1.0 |  |  |  | 1.0 |  |  |  | 1.0 |  |  |  | 1.0 |  |  |  | 1.0 |  |  |  |
| Unknown | 1.5 | 0.7 | 3.3 | 0.270 | 1.4 | 0.6 | 3.5 | 0.448 | 1.0 | 0.5 | 2.0 | 0.891 | 1.1 | 0.4 | 2.8 | 0.859 | 1.1 | 0.5 | 2.3 | 0.903 |
| Annual household income |  |  |  |  |  |  |  |  |  |  |  |  |  |  |  |  |  |  |  |  |
| <$35,000 | **3.3** | **2.3** | **4.7** | **0.000** | **2.9** | **1.8** | **4.5** | **0.000** | **1.4** | **1.0** | **2.0** | **0.040** | 1.5 | 1.0 | 2.3 | 0.076 | 1.1 | 0.7 | 1.5 | 0.789 |
| $35,000 - $74,999 | **1.7** | **1.2** | **2.4** | **0.003** | **1.6** | **1.0** | **2.6** | **0.042** | 1.1 | 0.8 | 1.5 | 0.620 | 1.2 | 0.8 | 1.8 | 0.332 | 0.9 | 0.6 | 1.2 | 0.359 |
| $75,000 | 1.0 |  |  |  | 1.0 |  |  |  | 1.0 |  |  |  | 1.0 |  |  |  | 1.0 |  |  |  |
| Current smoking prior to illness | **2.4** | **1.5** | **3.8** | **0.000** | 1.2 | 0.6 | 2.4 | 0.573 | 1.6 | 1.0 | 2.5 | 0.064 | 1.5 | 0.8 | 2.7 | 0.233 | 1.1 | 0.6 | 1.8 | 0.817 |
| Body Mass Index (BMI) |  |  |  |  |  |  |  |  |  |  |  |  |  |  |  |  |  |  |  |  |
| Underweight/normal weight (BMI < 25) | 1.0 |  |  |  | 1.0 |  |  |  | 1.0 |  |  |  | 1.0 |  |  |  | 1.0 |  |  |  |
| Overweight (BMI 25 to < 30) | 1.1 | 0.8 | 1.7 | 0.484 | 1.5 | 0.9 | 2.4 | 0.155 | 1.4 | 1.0 | 1.9 | 0.067 | 1.2 | 0.8 | 1.9 | 0.405 | 1.2 | 0.9 | 1.7 | 0.278 |
| Moderately obese (BMI 30 to <35) | **1.6** | **1.1** | **2.4** | **0.025** | **2.2** | **1.3** | **3.7** | **0.004** | 1.4 | 0.9 | 2.0 | 0.102 | 1.6 | 1.0 | 2.6 | 0.077 | 1.0 | 0.6 | 1.5 | 0.953 |
| Severely obese (BMI 35+) | **2.1** | **1.4** | **3.2** | **0.000** | **2.1** | **1.2** | **3.6** | **0.009** | 1.4 | 1.0 | 2.1 | 0.087 | 1.3 | 0.8 | 2.3 | 0.259 | 1.0 | 0.7 | 1.6 | 0.960 |
| Unknown | 2.1 | 0.6 | 7.7 | 0.267 | 2.7 | 0.6 | 12.3 | 0.208 | 1.6 | 0.4 | 5.8 | 0.482 | 1.8 | 0.4 | 8.5 | 0.465 | 1.5 | 0.4 | 5.5 | 0.512 |
| Any pre-existing physical condition (excluding psychological) | **1.9** | **1.5** | **2.6** | **0.000** | **1.8** | **1.3** | **2.7** | **0.001** | **1.3** | **1.0** | **1.7** | **0.060** | **1.8** | **1.3** | **2.6** | **0.001** | 0.9 | 0.7 | 1.2 | 0.372 |
| A pre-existing psychological condition | **4.9** | **3.2** | **7.6** | **0.000** | **2.1** | **1.2** | **3.7** | **0.009** | **2.5** | **1.6** | **3.8** | **0.000** | 1.6 | 0.9 | 2.9 | 0.111 | 0.9 | 0.5 | 1.7 | 0.796 |
| Phase |  |  |  |  |  |  |  |  |  |  |  |  |  |  |  |  |  |  |  |  |
| June 1, 2020 – September 30, 2020 | 1.0 |  |  |  | 1.0 |  |  |  | 1.0 |  |  |  | 1.0 |  |  |  | 1.0 |  |  |  |
| October 1, 2020 – February 28, 2021 | 1.0 | 0.6 | 1.7 | 0.946 | **0.5** | **0.3** | **0.9** | **0.031** | 1.4 | 0.9 | 2.3 | 0.156 | 0.9 | 0.5 | 1.6 | 0.690 | 0.9 | 0.5 | 1.4 | 0.590 |
| March 1, 2021 – May 31, 2021 | **1.7** | **1.0** | **3.0** | **0.047** | 1.0 | 0.5 | 1.8 | 0.882 | 1.6 | 0.9 | 2.7 | 0.093 | 0.7 | 0.3 | 1.4 | 0.310 | 1.1 | 0.6 | 1.9 | 0.823 |
| June 1, 2021 – September 30, 2021 | 1.1 | 0.6 | 2.0 | 0.690 | 0.7 | 0.4 | 1.4 | 0.335 | 1.5 | 0.9 | 2.5 | 0.143 | 1.1 | 0.6 | 2.1 | 0.820 | 1.4 | 0.8 | 2.5 | 0.206 |
| October 1, 2021 – February 28, 2022 | 1.5 | 0.9 | 2.4 | 0.132 | 0.7 | 0.4 | 1.2 | 0.194 | 1.5 | 0.9 | 2.5 | 0.098 | 0.9 | 0.5 | 1.6 | 0.670 | 1.2 | 0.7 | 2.0 | 0.499 |
| March 1, 2022 – May 31, 2022 | 0.7 | 0.4 | 1.2 | 0.188 | **0.5** | **0.2** | **0.9** | **0.022** | 1.3 | 0.8 | 2.1 | 0.352 | 0.8 | 0.4 | 1.4 | 0.410 | 1.5 | 0.9 | 2.5 | 0.098 |
| Survey mode |  |  |  |  |  |  |  |  |  |  |  |  |  |  |  |  |  |  |  |  |
| Phone | **1.5** | **1.1** | **2.0** | **0.006** | 1.2 | 0.8 | 1.7 | 0.348 | 0.9 | 0.6 | 1.1 | 0.265 | 1.2 | 0.8 | 1.7 | 0.430 | **0.7** | **0.5** | **1.0** | **0.028** |
| Online | 1.0 |  |  |  | 1.0 |  |  |  | 1.0 |  |  |  | 1.0 |  |  |  | 1.0 |  |  |  |

OR = odds ratio; LB=lower bound of 95% confidence interval; UB=upper bound of 95% confidence interval

Appendix Table 2. Acute COVID-19 symptom clusters excluding all individuals with less than 90 days between their COVID onset date and survey date, Michigan COVID-19 Recovery Surveillance Study (June 1, 2020 - May 31, 2022)

|  | **Cluster 1:  Multi-symptomatic** | **Cluster 2:  Predominance of shortness of breath** | **Cluster 3:  Flu-like** | **Cluster 4:  Predominance of fatigue** | **Cluster 5:  Fever** | **Cluster 6:  Nasal congestion** |
| --- | --- | --- | --- | --- | --- | --- |
| Fatigue | 1.00 | 0.97 | 0.99 | 0.98 | 0.86 | 0.43 |
| Felt feverish or fever over 100.4F/38C | 0.92 | 0.83 | 0.89 | 0.18 | 0.80 | 0.12 |
| Chills or repeated shaking with chills | 0.94 | 0.84 | 0.92 | 0.00 | 0.74 | 0.09 |
| Muscle aches | 0.98 | 0.78 | 0.96 | 0.65 | 0.63 | 0.14 |
| Muscle weakness or general weakness | 0.99 | 0.77 | 0.85 | 0.67 | 0.42 | 0.07 |
| Joint pain | 0.72 | 0.25 | 0.43 | 0.25 | 0.09 | 0.00 |
| Shortness of breath | 0.91 | 0.97 | 0.33 | 0.41 | 0.20 | 0.07 |
| Cough | 0.92 | 0.80 | 0.62 | 0.52 | 0.50 | 0.28 |
| Nasal congestion or runny nose | 0.87 | 0.72 | 0.76 | 0.67 | 0.65 | 0.58 |
| Sore throat | 0.74 | 0.51 | 0.56 | 0.35 | 0.38 | 0.29 |
| Nausea or vomiting, abdominal pain, or diarrhea | 0.85 | 0.40 | 0.51 | 0.37 | 0.21 | 0.08 |
| Loss of appetite | 0.86 | 0.55 | 0.66 | 0.48 | 0.34 | 0.10 |
| Headache | 0.96 | 0.68 | 0.89 | 0.67 | 0.57 | 0.30 |
| Lightheaded or dizzy | 0.89 | 0.46 | 0.47 | 0.29 | 0.08 | 0.06 |
| Brain fog, memory loss, disorientation | 0.72 | 0.38 | 0.39 | 0.33 | 0.10 | 0.04 |
| Loss of sense of smell or taste | 0.78 | 0.57 | 0.61 | 0.63 | 0.51 | 0.38 |
| Chest pain or tightness | 0.76 | 0.56 | 0.12 | 0.20 | 0.06 | 0.06 |
| Heart rate or rhythm issues | 0.35 | 0.17 | 0.04 | 0.08 | 0.01 | 0.01 |
| Hair loss | 0.20 | 0.03 | 0.07 | 0.06 | 0.01 | 0.02 |
| Rash or skin discoloration | 0.12 | 0.04 | 0.03 | 0.02 | 0.01 | 0.02 |
| **Prevalence in the sample** | **14.7** | **10.5** | **24.3** | **10.6** | **23.8** | **16.1** |

Values for symptoms represent the probability of individuals in each cluster reporting each symptom

Appendix Table 3. Modified Poisson regression examining prevalence of Long COVID excluding all individuals with less than 90 days between their COVID onset date and survey date (n=3639), Michigan COVID-19 Recovery Surveillance Study (June 1, 2020 - May 31, 2022)

|  | Unadjusted | | | | Adjusted | | | |
| --- | --- | --- | --- | --- | --- | --- | --- | --- |
|  | PR | 95 % CI | | p value | aPR | 95 % CI | | p value |
|  |  | LB | UB |  |  | LB | UB |  |
| Acute COVID-19 symptom clusters |  |  |  |  |  |  |  |  |
| Multi-symptomatic | **6.5** | **4.6** | **9.2** | **0.000** | **5.6** | **4.0** | **7.9** | **0.000** |
| Predominance of shortness of breath | **3.7** | **2.5** | **5.4** | **0.000** | **3.5** | **2.4** | **5.1** | **0.000** |
| Flu-like | **2.7** | **1.9** | **3.9** | **0.000** | **2.7** | **1.9** | **3.8** | **0.000** |
| Predominance of fatigue | **2.6** | **1.7** | **3.9** | **0.000** | **2.2** | **1.5** | **3.3** | **0.000** |
| Fever | 1.1 | 0.7 | 1.7 | 0.695 | 1.2 | 0.8 | 1.8 | 0.461 |
| Nasal congestion | 1.0 |  |  |  | 1.0 |  |  |  |
| Sex |  |  |  |  |  |  |  |  |
| Male | 1.0 |  |  |  | 1.0 |  |  |  |
| Female | **1.7** | **1.5** | **2.0** | **0.000** | **1.4** | **1.2** | **1.7** | **0.000** |
| Age group |  |  |  |  |  |  |  |  |
| 18-34 | 1.0 |  |  |  | 1.0 |  |  |  |
| 35-44 | **1.9** | **1.5** | **2.4** | **0.000** | **1.8** | **1.4** | **2.2** | **0.000** |
| 45-54 | **1.9** | **1.5** | **2.4** | **0.000** | **1.7** | **1.4** | **2.2** | **0.000** |
| 55-64 | **2.0** | **1.6** | **2.5** | **0.000** | **2.1** | **1.6** | **2.6** | **0.000** |
| 65+ | **2.4** | **1.9** | **3.0** | **0.000** | **2.5** | **2.0** | **3.2** | **0.000** |
| Race/ethnicity |  |  |  |  |  |  |  |  |
| Hispanic | **1.4** | **1.1** | **1.9** | **0.006** | 1.1 | 0.9 | 1.4 | 0.423 |
| Non-Hispanic Black | **1.4** | **1.1** | **1.8** | **0.008** | 1.1 | 0.9 | 1.4 | 0.377 |
| Another race/ethnicity | 0.8 | 0.6 | 1.1 | 0.199 | 0.8 | 0.6 | 1.0 | 0.093 |
| Non-Hispanic white | 1.0 |  |  |  | 1.0 |  |  |  |
| Unknown | 0.9 | 0.6 | 1.4 | 0.692 | 0.9 | 0.6 | 1.5 | 0.760 |
| Annual household income |  |  |  |  |  |  |  |  |
| <$35,000 | **1.6** | **1.3** | **1.9** | **0.000** | **1.2** | **1.0** | **1.5** | **0.032** |
| $35,000 - $74,999 | **1.4** | **1.1** | **1.6** | **0.001** | 1.2 | 1.0 | 1.4 | 0.057 |
| $75,000 | 1.0 |  |  |  | 1.0 |  |  |  |
| Current smoking prior to illness | **1.3** | **1.0** | **1.6** | **0.020** | 1.1 | 0.9 | 1.4 | 0.385 |
| Body Mass Index (BMI) |  |  |  |  |  |  |  |  |
| Underweight/normal weight (BMI < 25) | 1.0 |  |  |  | 1.0 |  |  |  |
| Overweight (BMI 25 to < 30) | **1.3** | **1.0** | **1.6** | **0.026** | 1.2 | 0.9 | 1.4 | 0.184 |
| Moderately obese (BMI 30 to <35) | **1.6** | **1.3** | **2.0** | **0.000** | 1.2 | 1.0 | 1.5 | 0.117 |
| Severely obese (BMI 35+) | **2.0** | **1.6** | **2.5** | **0.000** | **1.4** | **1.1** | **1.7** | **0.005** |
| Unknown | 1.2 | 0.6 | 2.4 | 0.651 | 1.0 | 0.5 | 2.2 | 0.979 |
| Any pre-existing physical condition (excluding psychological) vs. none | **1.8** | **1.5** | **2.1** | **0.000** | **1.2** | **1.0** | **1.4** | **0.039** |
| A pre-existing psychological condition vs. none | **1.5** | **1.2** | **1.8** | **0.000** | 1.1 | 0.9 | 1.3 | 0.399 |
| Phase |  |  |  |  |  |  |  |  |
| June 1, 2020 – September 30, 2020 | 1.0 |  |  |  | 1.0 |  |  |  |
| October 1, 2020 – February 28, 2021 | 1.0 | 0.8 | 1.2 | 0.736 | 0.9 | 0.7 | 1.1 | 0.285 |
| March 1, 2021 – May 31, 2021 | 0.9 | 0.7 | 1.1 | 0.242 | **0.8** | **0.6** | **1.0** | **0.024** |
| June 1, 2021 – September 30, 2021 | 0.9 | 0.7 | 1.2 | 0.376 | 0.8 | 0.6 | 1.1 | 0.144 |
| October 1, 2021 – February 28, 2022 | 0.8 | 0.6 | 1.1 | 0.131 | **0.7** | **0.6** | **0.9** | **0.001** |
| March 1, 2022 – May 31, 2022 | **0.5** | **0.3** | **0.6** | **0.000** | **0.5** | **0.4** | **0.7** | **0.000** |
| Survey mode |  |  |  |  |  |  |  |  |
| Phone | **1.4** | **1.2** | **1.7** | **0.000** | **1.2** | **1.0** | **1.4** | **0.020** |
| Online | 1.0 |  |  |  | 1.0 |  |  |  |

PR = prevalence ratio; aPR = adjusted prevalence ratio; LB=lower bound of 95% confidence interval; UB=upper bound of 95% confidence interval

^a^All models mutually adjusted for variables listed in table
